# The Role of ClpB in Bacterial Stress Responses and Virulence

**DOI:** 10.3389/fmolb.2021.668910

**Published:** 2021-04-22

**Authors:** Athar Alam, Jeanette E. Bröms, Rajender Kumar, Anders Sjöstedt

**Affiliations:** Laboratory for Molecular Infection Medicine Sweden (MIMS), Department of Clinical Microbiology, Umeå University, Umeå, Sweden

**Keywords:** ClpB chaperone, stress response, heat shock, type VI secretion, ClpB inhibitor

## Abstract

Bacterial survival within a mammalian host is contingent upon sensing environmental perturbations and initiating an appropriate counter-response. To achieve this, sophisticated molecular machineries are used, where bacterial chaperone systems play key roles. The chaperones are a prerequisite for bacterial survival during normal physiological conditions as well as under stressful situations, e.g., infection or inflammation. Specific stress factors include, but are not limited to, high temperature, osmolarity, pH, reactive oxidative species, or bactericidal molecules. ClpB, a member of class 1 AAA^+^ proteins, is a key chaperone that via its disaggregase activity plays a crucial role for bacterial survival under various forms of stress, in particular heat shock. Recently, it has been reported that ClpB also regulates secretion of bacterial effector molecules related to type VI secretion systems. In this review, the roles of ClpB in stress responses and the mechanisms by which it promotes survival of pathogenic bacteria are discussed.

## Introduction and Overview

Upon infection of a host, most bacterial pathogens experience drastic changes in their environment, e.g., with regard to pH, temperature and osmolarity. In addition, host inflammatory responses recruit phagocytic cells, subjecting pathogens to additional adverse conditions, such as oxidative and nitrosative stresses. Bacterial survival then depends on molecular adaptations, so called stress responses, to handle the adverse conditions. Essential to these responses are the heat shock proteins (Hsps) which act as molecular chaperones to stabilize proteins and assist protein refolding under stressful conditions (Neckers and Tatu, 2008). DnaJ (Hsp40), GroEL (Hsp60), DnaK (Hsp70), HtpG (Hsp90), and ClpB (Hsp100) are some of the major bacterial molecular chaperones that function in cooperation by forming complex molecular networks, thereby maintaining the overall cellular protein homeostasis (Henderson et al., 2006).

ClpB is a member of the AAA^+^ family (ATPases associated with diverse cellular activities) that together with the DnaK system have the ability to disaggregate stress-denatured proteins. Like other members of the Hsp100 family, ClpB constitutes a hexamer of identical monomers. The monomer of ClpB comprises four domains: an N-terminal domain connected with the remainder of the protein by a conserved linker, the first nucleotide binding domain (NBD-1) in which the unique flexible middle (M) domain is located, and a second NBD (NBD-2) (Lee et al., 2003). Translocation of unfolded protein substrates through the axial protein channel requires that NBD-1 and −2 must couple their ATPase activity (Deville et al., 2017). The M-domain is involved in the direct interaction of ClpB with DnaK (Haslberger et al., 2007), in the interaction of the monomer with neighboring ClpB monomers via their NBD-1 domains (Oguchi et al., 2012), and in the stabilization of the hexamer (del Castillo et al., 2011).

ClpB is highly conserved amongst bacteria, fungi, protozoa, and plants and its role under different stressful conditions has been much studied. It provides protection against, e.g., heat, low pH, osmotic- and oxidative stress, ethanol, and nutrient starvation (Meibom et al., 2008; Krajewska et al., 2017; Glaza et al., 2020; Tripathi et al., 2020). Thus, *clpB*-deficient mutants demonstrate tremendously decreased survival upon exposure to these stresses. Furthermore, ClpB has also been implicated to regulate the expression of virulence factors in several pathogenic bacteria (Frees et al., 2004; Yuan et al., 2007; Capestany et al., 2008; de Oliveira et al., 2011; Lourdault et al., 2011; Alam et al., 2018; Sangpuii et al., 2018). Therefore, ClpB is critical for survival and infectivity of a broad range of clinically relevant microorganisms.

In addition to its role in solubilizing stress-induced protein aggregates, a role of ClpB in type VI secretion (T6S) has recently been reported in the highly pathogenic bacterium *Francisella tularensis* (Brodmann et al., 2017; Alam et al., 2018, 2020). Here, ClpB apparently serves as a functional homolog of ClpV, harnessing energy through the hydrolysis of ATPs required for depolymerization of the IglA-IglB (homologs of *Vibrio cholerae* VipA-VipB) sheath for recycling and reassembly. Consequently, deletion of *clpB* leads to significantly reduced level of T6S and complete attenuation of *F. tularensis* in mice (Alam et al., 2018, 2020).

Molecular chaperones have the potential to serve as critical targets for the development of novel antimicrobials. For example, the Hsp70 and Hsp90 ATPases have been identified as drug targets for protozoan-derived infectious diseases in humans (Zininga and Shonhai, 2014, 2019). However, due to the high degree of sequence conservation among the Hsps across different domains of life, it is a challenging task (Glaza et al., 2020). ClpB is of special relevance as a drug target, since the homolog of ClpB, Skd3, also known as human ClpB, is conserved in many metazoan lineages, but differs significantly from bacterial and yeast proteins in domain structures. Skd3 lacks the characteristic microbial ClpB coiled-coil domain and contains a unique ankyrin-repeat domain (Erives and Fassler, 2015; Cupo and Shorter, 2020). In contrast, eubacteria and non-metazoan eukaryotes harbor Hsp104, which is more closely related to microbial ClpB (Oguchi et al., 2012).

This review aims to elucidate our current understanding of the ClpB chaperones of pathogenic bacteria and their potential contribution to virulence. Since ClpB affects infectivity and survival of a broad range of clinically relevant pathogenic microorganisms, the possibility of exploiting ClpB as a therapeutic target is also discussed.

## The Role of ClpB in Stress-Tolerance and Virulence

**FIGURE 1:**
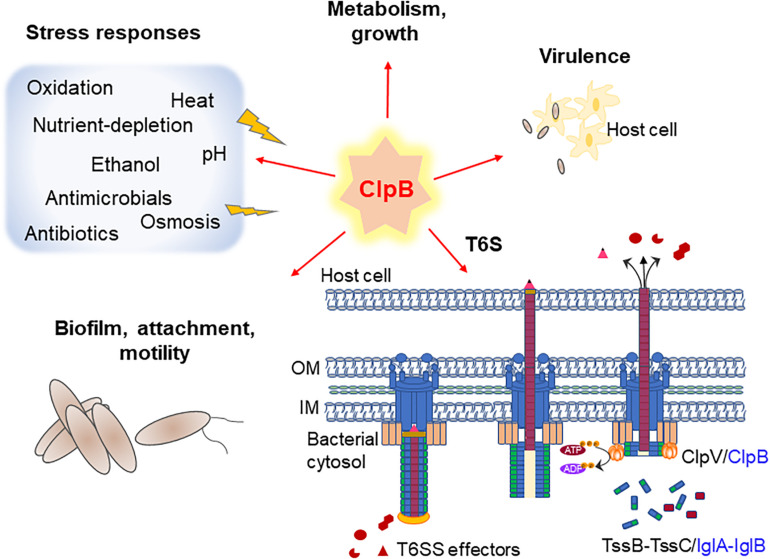
A summary of ClpB’s so-far established roles in pathogenic bacteria, including T6S (*Francisella* only). Schematic figure illustrating the importance of ClpB in various stress responses, T6S and virulence. Model of the T6S in extended (left), contracted (central), and disassembled (right) forms of canonical and *Francisella* T6S is shown, where ClpB acts as an energizer. Canonical T6S subunits from *Escherichia coli* are labeled in black and *Francisella* T6S subunits, which are encoded within the *Francisella* Pathogenicity Island (FPI), are labeled in blue.

One of the fundamental roles of ClpB is to mediate tolerance to stressful conditions, in particular heat, for a wide range of bacterial species (Figure 1 and Table 1), but if and how ClpB contributes to bacterial survival during infection has been less studied. The *Escherichia coli* ClpB has served as the prototype for studies of the essential mechanisms of Hsp100 disaggregases during heat shock and for the structural identification of the various domains (Squires et al., 1991; Mogk et al., 1999, 2015; Barnett et al., 2000; Rosenzweig et al., 2013). Due to the high degree of conservation among bacterial ClpB, the *E. coli* ClpB data is often being used to infer the structures and roles of ClpB proteins of other bacterial species.

**TABLE 1:** The impact of the chaperone ClpB on bacterial growth, survival, and virulence in various bacterial species.

	Role of ClpB in bacterial stress responses^1^				
Species	Heat shock	Other stresses	Additional observations for *clpB* mutants	Attenuation in indicated host model or cell infection	References
*Acinetobacter baumannii*	NT	S (beta-lactams)			Lazaretti et al., 2020
*Brucella suis*	S	S (ethanol, pH)			Ekaza et al., 2001
*Campylobacter coli, lari*	S	NT			Riedel et al., 2020
*Ehrlichia chaffeensis*	T	NT		DH82 canine macrophage cell line	Grimminger et al., 2004; Zhang et al., 2013
*Enterococcus faecalis*	S	T (oxidative stress)		*Galleria mellonella*	de Oliveira et al., 2011
*Escherichia coli*	S	S (oxidative stress)			Chow and Baneyx, 2005; Martin et al., 2013
*Francisella noatunensis*	NT	NT		Zebrafish	Lampe et al., 2017
*Francisella novicida*	S	NT	↓ T6S	J774 cell line, Mice	Alam et al., 2020
*Francisella tularensis* subsp. *tularensis*	S	S (pH)	↓ T6S	Mice	Alam et al., 2018
*Francisella tularensis* subsp. *holarctica*	S	S (oxidative stress, pH, ethanol	↓ T6S	Mice	Meibom et al., 2008; Alam et al., 2018
*Helicobacter pylori*	S	NT			Allan et al., 1998
*Leptospira interrogans*	S	S (oxidative stress) S (nutrient-limitation)		Gerbil	Lourdault et al., 2011; Kêdzierska-Mieszkowska and Arent, 2020
*Listeria monocytogenes*	T	T (osmosis/salt) T (cold)		Mice	Chastanet et al., 2004
*Mycobacterium tuberculosis*	NT	S (oxidative stress)	Aberrant cellular morphology, impaired biofilm formation and defective maintenance of dormant bacteria	THP-1 cell line	Harnagel et al., 2020; Singh et al., 2020; Tripathi et al., 2020
*Mycoplasma pneumoniae*	NT	NT	Impaired growth under permissive conditions.		Kannan et al., 2008
*Piscirickettsia salmonis*	NT	NT		SHK-1 salmon cell line	Isla et al., 2014
*Porphyromonas gingivalis*	S	T (pH) T (oxidative stress)		Mice; Gingival epithelial cells and Human coronary artery endothelial cells	Yuan et al., 2007; Capestany et al., 2008
*Pseudomonas putida*	S	NT			Ito et al., 2014
*Pseudomonas aeruginosa*	NT	S (tobramycin)			Wu et al., 2015
*Salmonella typhimurium*	S	S (oxidative stress)		Chicken	Sangpuii et al., 2018
*Staphylococcus aureus*	S	T (oxidative stress)		MAC-T bovine mammary epithelial cell line	Frees et al., 2004
*Vibrio cholerae*	S	S (pH) S (oxidative stress)			Nag et al., 2005
*Yersinia enterocolitica*	NT	NT	↓ Invasin expression ↓ Motility		Badger et al., 2000

*^1^Abbreviations used are as follows: S, sensitive; T, tolerant; NT, not tested. ↓Decreased.*

*E. coli, F. tularensis*, *Helicobacter pylori, Pseudomonas putida, Campylobacter coli*, and *Campylobacter lari* are some of the pathogenic bacteria for which the role of ClpB in thermotolerance has been studied (Squires et al., 1991; Allan et al., 1998; Meibom et al., 2008; Ito et al., 2014; Alam et al., 2018, 2020; Riedel et al., 2020). *F. tularensis*, a highly infectious pathogen and a category A bioterrorism agent, is the etiological agent of the zoonotic disease tularemia. Deletion of the *clpB* gene causes a severe defect in survival at elevated temperature (Meibom et al., 2008; Alam et al., 2018, 2020). A similar effect was observed for a *clpB* mutant of *H. pylori*, the causative agent of gastric ulcers (Allan et al., 1998). In addition to thermosensitivity, an inability to disaggregate aggregated proteins was demonstrated for a *clpB* mutant of the opportunistic human pathogen *P. putida* (Ito et al., 2014; Table 1). Moreover, enhanced levels of *clpB* gene expression were observed at elevated temperature in *Campylobacter*, a genus containing one of the most important food-borne pathogen globally. Transcriptomic profiles of *C. coli* and *C. lari* at elevated temperatures showed enhanced gene expression of *clpB* and other genes encoding chaperones such as *dnaK*, *groES*, and *groEL*, indicating that multiple chaperones, including ClpB, play a vital role in the thermotolerance of *Campylobacter* spp. (Riedel et al., 2020).

In addition to its importance for thermotolerance, ClpB also plays a role in the general stress-tolerance of bacteria (Figure 1 and Table 1). A *clpB* null mutant of *Brucella suis*, the etiological agent of swine brucellosis, showed increased sensitivity not only to high temperature, but also to ethanol and acid pH (Ekaza et al., 2001). A specific role of ClpB during antibiotic-induced stress has also been reported in *Acinetobacter baumannii*, a multi-resistant, opportunistic human pathogen. Levels of *clpB* were dramatically increased in the presence of the carbapenem meropenem, or trimethoprim/sulfamethoxazole, indicating that the chaperone may play a key role for antibiotic resistance (Lazaretti et al., 2020). Similarly, inactivation of *ibpA/clpB* increased the susceptibility to the aminoglycoside tobramycin in the opportunistic human pathogen *P. aeruginosa* (Wu et al., 2015; Table 1).

Besides promoting stress tolerance, ClpB plays an important role in invasiveness and/or host survival of multiple important bacterial pathogens (Table 1), such as *Leptospira interrogans, Yersinia enterocolitica, Francisella noatunensis, F. tularensis, Piscirickettsia salmonis, Mycoplasma pneumoniae, Salmonella typhimurium, Mycobacterium tuberculosis, Porphyromonas gingivalis, Enterococcus faecalis, Listeria monocytogenes*, and *Staphylococcus aureus* (Badger et al., 2000; Chastanet et al., 2004; Frees et al., 2004; Yuan et al., 2007; Capestany et al., 2008; Kannan et al., 2008; Conlan, 2011; de Oliveira et al., 2011; Lourdault et al., 2011; Alam et al., 2018, 2020; Sangpuii et al., 2018; Harnagel et al., 2020; Kêdzierska-Mieszkowska and Arent, 2020; Tripathi et al., 2020). In the case of *L. interrogans*, the causative agent of the emerging zoonotic disease leptospirosis, a *clpB* mutant not only showed enhanced susceptibility to high temperature, nutrient-depletion, and oxidative stress, but was also attenuated in a gerbil animal model of acute leptospirosis (Lourdault et al., 2011; Kêdzierska-Mieszkowska and Arent, 2020). Similarly, a *Y. enterocolitica clpB* mutant demonstrated defective invasion of human laryngeal epithelial cells, Hep-2, and reduced expression of important virulence factors, including invasin and flagellin (Badger et al., 2000). *F. noatunensis* ssp. *noatunensis* is the etiological agent of francisellosis in Atlantic cod. In the absence of ClpB, the resulting mutant showed attenuation in a zebrafish model and also provided efficient protection in zebrafish challenged with wild-type bacteria (Lampe et al., 2017). Moreover, *clpB* mutants of *F. tularensis* subspecies *holarctica* and *tularensis* were found to be defective for T6S, susceptible to elevated temperature, and completely attenuated in mice (Alam et al., 2018, 2020). Such mutants also serve as highly efficacious vaccines in animal models of tularemia (Conlan, 2011; Alam et al., 2018). *P. salmonis*, the etiological agent of salmonid rickettsial septicemia (SRS), a disease that affects a wide variety of cultivated fish species, demonstrated significantly higher levels of ClpB during intramacrophage growth in a salmon cell line; indicating that this permits the pathogen to adapt to the hostile intracellular conditions and facilitates replication (Isla et al., 2014). A growth-promoting status of ClpB was also observed in *M. pneumoniae*, an important cause of community-acquired pneumonia, since loss of ClpB resulted in impaired replication under permissive growth conditions (Kannan et al., 2008). ClpB also plays a vital role in the survival in chicken of *S. typhimurium*, a major cause of gastroenteritis globally, since a *clpB* mutant was found to display reduced survival at 42°C in poultry macrophages and during exposure to hypochloric acid and paraquat (Sangpuii et al., 2018). Moreover, the mutant showed decreased dissemination *in vivo* (Sangpuii et al., 2018). *M. tuberculosis*, the causative agent of tuberculosis, is one of the most important pathogens globally. It was demonstrated that a *clpB* mutant of *M. tuberculosis* has aberrant cellular morphology, impaired biofilm formation and reduced cellular infectivity (Tripathi et al., 2020). In addition, the mutant was sensitive to oxidative stress and defective for the maintenance of dormant bacteria (Harnagel et al., 2020; Tripathi et al., 2020). Furthermore, the purified ClpB protein from *M. tuberculosis* showed potent biological activity and induced release of pro-inflammatory cytokines from a human macrophage cell line (Tripathi et al., 2020). A vital role of ClpB was also observed in *P. gingivalis*, an important cause of chronic periodontal disease, where a *clpB* mutant showed defective thermotolerance and also decreased cellular invasion and marked attenuation in a mouse model (Yuan et al., 2007; Capestany et al., 2008). ClpB of the Gram-positive bacterium *L. monocytogenes*, an etiological agent of human meningitis, was not involved in tolerance to heat, high salt, or cold; but played a role for virulence in mice (Chastanet et al., 2004). *S. aureus*, a major cause of skin infections and several systemic infections, was susceptible to elevated heat stress and a *clpB* mutant demonstrated diminished intracellular multiplication within bovine mammary epithelial cells (Frees et al., 2004). Similarly, *E. faecalis* lacking ClpB demonstrated defective thermotolerance, as well as attenuation in a Galleria mellonella model (de Oliveira et al., 2011). Altogether, the published data unequivocally demonstrate that ClpB of many bacterial species play a key role for their survival during numerous forms of stress conditions and for their virulence in experimental models.

## The Role of ClpB for T6S

The type VI secretion systems comprise the most common secretion machinery among Gram-negative bacteria, present in more than 25% of all proteobacteria. T6S is used to translocate effector molecules directly into neighboring cells, commonly a bacterial competitor (Coulthurst, 2019). The machinery is composed of 13 to 14 core components, with a set of regulatory and accessory proteins for specialized functions (Boyer et al., 2009). It is composed of a cell membrane complex anchored to a contractile bacteriophage tail-like apparatus consisting of a sharpened tube made of stacked hexameric rings ejected by the contraction of a sheath (Coulthurst, 2019). The AAA^+^ ATPase ClpV has been shown to act as an energizer for T6S. Its action includes physical interactions with the complexes of VipA-VipB, or their homologs, of the contracted tubular sheath, thereby promoting sheath disassembly and the dynamic recycling for repeated rounds of firing, disassembly and reassembly (Figure 1; Bönemann et al., 2009; Pietrosiuk et al., 2011; Kube et al., 2014). For some bacteria, the energy may be provided through the activity of ATPases distinct to ClpV, which are encoded outside of the T6S cluster. In support, only a partial loss of the function of T6S was observed in a *V. cholerae clpV* mutant, demonstrating that ClpV is an important, yet non-essential component of the *V. cholerae* T6S (Basler and Mekalanos, 2012; Basler et al., 2012). Moreover, *Francisella* spp., *Campylobacter concisus*, *Campylobacter jejuni*, *Helicobacter hepaticus*, and *Salmonella choleraesuis*, all lack ClpV, but still possess functional T6S (Shrivastava and Mande, 2008; Lertpiriyapong et al., 2012; Clemens et al., 2015; Brodmann et al., 2017; Liaw et al., 2019; Alam et al., 2020; Liu et al., 2020).

Indeed, Brodmann et al. (2017) have demonstrated that ClpB, although encoded separately from the T6S system gene cluster in *Francisella*, is a functional homolog of ClpV in *F. tularensis*, being indispensable for disassembly of the contracted T6S system sheath (Figure 1) and important for effective T6S (Alam et al., 2018, 2020). Moreover, ClpB was shown to colocalize with the VipA homolog, IglA, during sheath assembly, contraction, and disassembly (Brodmann et al., 2017). Interestingly, a conserved α-helical region at the N-terminus of VipB, including the part interacting with ClpV, is missing in the *F. tularensis* homolog IglB (Pietrosiuk et al., 2011), but, despite a very low overall sequence identity, IglB and VipB share a very similar structural topology (Alam et al., 2020). Though the sheath sequence(s) recognized by ClpB ATPase has not been determined, the overall similar topology may be sufficient for establishing the interaction. Interestingly, a *clpB* mutation that abolishes the ClpB-DnaK interaction renders *F. tularensis* highly susceptible to heat shock, but T6S and virulence in mice are unaffected (Alam et al., 2020). This suggests that the heat shock response and the regulation of T6S of *F. tularensis* are dependent on distinct regions of the ClpB protein and that the DnaK interaction is dispensable for T6S (Alam et al., 2020). ClpB-dependent secretion mechanisms could perhaps be at play also in the aforementioned species possessing functional T6S, but lacking ClpV; however, the contribution of ATPases distinct from ClpB cannot be excluded. Notably, in the malaria parasite, a ClpB-like protein of the Hsp101 family is essential for export across the parasitophorous vacuolar membrane into the erythrocyte and it was demonstrated that the protein functions in a complex that serves as a convergent step in a multi-pathway export process (Beck et al., 2014).

## ClpB as a Therapeutic Target

The global threat of antibiotic-resistant bacteria shows no sign of being resolved and the arsenal of clinically useful antibiotics becomes more and more limited. Bacterial chaperones remain one set of underexploited targets for antibiotic development. In particular, ClpB belongs to the group of potential drug targets, since mammals do not have Hsp100 homologs, other than human ClpB/Skd3 which is significantly different from the microbial ClpB in domain structures (Erives and Fassler, 2015; Cupo and Shorter, 2020). The development of specific inhibitors of ClpB might not only be useful as a novel antibiotic for otherwise antibiotic-resistant bacterial strains, but also as a means to understand the molecular mechanism of this chaperone.

Currently, only a few ClpB inhibitors have been identified (Grimminger et al., 2004; Martin et al., 2013; Kuczynska-Wisnik et al., 2017; Glaza et al., 2020; Singh et al., 2020). Guanidinium chloride specifically inhibits the ATP hydrolysis by Hsp104 of *Saccharomyces cerevisiae* and also the ClpB function of *Ehrlichia chaffeensis* (Grimminger et al., 2004). Thus, it may serve as a general inhibitor of members of the AAA^+^ protein family, but this remains to be proven. Two other ClpB inhibitors, called compounds 3 and 6, inhibit the functional properties and the growth of *E. coli*, thus displaying antimicrobial activity under thermal or oxidative stress conditions (Martin et al., 2013). Compound 3 competes with substrate binding and modifies the ATPase activity of ClpB, while compound 6 hampers the substrate-induced improvement of its ATPase activity (Martin et al., 2013). Further, the specific interaction of the compounds with the chaperone is essential for their antimicrobial action. This, in combination with only moderate cytotoxicity, suggests that they could be used as leads for development of new antimicrobials (Martin et al., 2013). Three inhibitors of *M. tuberculosis* ClpB have been identified and they also inhibit the ATPase activity of *E. coli* ClpB and yeast Hsp104 (Singh et al., 2020). In addition, DBeQ, which is derived from an inhibitor of the human AAA^+^ ATPase p97, an anti-tumor target, inhibited *E. coli* proliferation and appeared to selectively target ClpB (Glaza et al., 2020).

Collectively, the identification of these ClpB inhibitors demonstrates the potential of the protein as a therapeutic target.

## Conclusion

The ATP-dependent ClpB protein is a disaggregase and a key member of a multi-chaperone system that efficiently inhibits and reverses protein aggregation. As such, ClpB is critical for the survival of various microorganisms exposed to stress, but it also confers vital functions during normal physiological conditions. In bacteria, loss of ClpB is commonly associated with fatal thermosensitivity, but it may also lead to susceptibility to other forms of stress, such as reactive oxidative species, antibiotics and bactericidal molecules as well as changes in osmolarity and pH. More recent work has identified a critical role of ClpB related to T6S. Thus, in *F. tularensis*, the absence of ClpB leads to T6S dysfunction and impaired bacterial virulence. This also suggests that the ATPase activity of ClpB may provide the energy required for functional T6S, thereby substituting for ClpV proteins in bacteria where these are absent. In view of the many central roles of ClpB, it is a logical therapeutic target and recent work serves as proof of concept for this hypothesis.

## Author Contributions

AA, JEB, and AS conceptualized the manuscript, involved in the generation of the figure, and critically revised the manuscript. AA, JEB, RK, and AS wrote the manuscript. All authors have read the article and approved it for publication.

## Conflict of Interest

The authors declare that the research was conducted in the absence of any commercial or financial relationships that could be construed as a potential conflict of interest.
